# Role of Oxidative Stress and Mitochondrial Dysfunction in Sepsis and Potential Therapies

**DOI:** 10.1155/2017/5985209

**Published:** 2017-08-20

**Authors:** Konstantinos Mantzarlis, Vasiliki Tsolaki, Epaminondas Zakynthinos

**Affiliations:** Department of Critical Care, University Hospital of Larissa, University of Thessaly Medical School, Larissa, Thessaly, Greece

## Abstract

Sepsis is one of the most important causes of death in intensive care units. Despite the fact that sepsis pathogenesis remains obscure, there is increasing evidence that oxidants and antioxidants play a key role. The imbalance of the abovementioned substances in favor of oxidants is called oxidative stress, and it contributes to sepsis process. The most important consequences are vascular permeability impairment, decreased cardiac performance, and mitochondrial malfunction leading to impaired respiration. Nitric oxide is perhaps the most important and well-studied oxidant. Selenium, vitamin C, and 3N-acetylcysteine among others are potential therapies for the restoration of redox balance in sepsis. Results from recent studies are promising, but there is a need for more human studies in a clinical setting for safety and efficiency evaluation.

## 1. Introduction

Sepsis is the leading cause of mortality in the intensive care units [1, 2]. Recent publications regarding the definition [3] and management of sepsis [4] underline the keen interest of clinicians. Despite the research, sepsis pathogenesis remains obscure. In the past, the widely accepted theory reported that sepsis was an uncontrolled inflammatory response to a pathogen that was rather a bystander than the real insult [5]. The failure of numerous studies using anti-inflammatory agents questioned the hypothesis of hyperinflammation [6–9].

Therapies focused until recently on macrocirculatory failure such as decreased mean arterial pressure and cardiac output. Immunohistohemical analysis revealed that cell death is minor suggesting that mechanisms other than cell death are responsible for mortality [10]. A growing body of evidence suggests that the inability of the cell to consume oxygen may play a crucial role for sepsis pathogenesis. For example, studies where supranormal oxygen delivery was targeted failed to improve patients' outcomes [11]. Furthermore, in animal studies, mucosal acidosis persisted despite the fact that mucosal perfusion did not change [12]. Since mitochondrial O_2_ consumption is 90% of the total body consumption, impaired O_2_ utilization and dysfunctional mitochondria may explain sepsis' specific characteristics. Sepsis is also characterized by excessive production of oxidants. Therefore, they may represent the generator of the abovementioned abnormalities that lead to increased mortality. In this context, redox homeostasis may play a key role, and consequently, therapies targeted to redox abnormalities may be useful for better management of septic patients.

Despite the increasing evidence that oxidative stress is a cornerstone on sepsis pathogenesis, the role of oxidative stress in sepsis may be underestimated. For example, in recent sepsis guidelines, its significance has not been highlighted. In this respect, clinicians may not be aware of the potentially pivotal role of oxidative stress in sepsis evolution. The aim of this literature review article is to point out current aspects about the topic and the evaluation of potential therapies.

## 2. Oxidants and Antioxidants

Redox reactions represent the basis for numerous biochemical mechanisms imperative for physiological cell function like cell signaling [13, 14]. Oxidants and antioxidants play a key role in the abovementioned mechanisms. The term antioxidant refers to a substance which donates electrons, whereas an oxidant is a substance that accepts electrons [15]. Oxidants are involved in the formation of deoxyribonucleotides, prostaglandin production, oxidation, and carboxylation and hydroxylation reactions that are essential for normal cell function. Free radicals also participate in the host defense against bacterial infections [16], the regulation of vascular tone, and cell adhesion reactions and act as a sensor for oxygen concentration [17]. Important reactive oxygen species (ROS) in sepsis pathogenesis include superoxide (O_2_^−^), hydrogen peroxide (H_2_O_2_), and hydroxyl radicals (HO). O_2_^−^ and HO are free radicals since they have unpaired electrons in their molecule. Reactive nitrogen species (RNS) include the free radical nitric oxide (NO) and the nonradical peroxynitrite (ONOO^−^). There are several procedures involved in the genesis of oxidant molecules in health [18] and sepsis. Cells that represent the innate immune system, like neutrophils and macrophages, are responsible for the oxidative burst that takes place early in sepsis process [19, 20]. The generated ROS and RNS are important for host defense as it was demonstrated by studies with mice deficient to produce O_2_^−^, a fact associated with decreased bacterial clearance [21]. The expression of nitric oxide synthase (NOS) is enhanced by lipopolysaccharide (LPS) treatment and nuclear factor kB (NF-kB) activation, and consequently, NO concentration produced by L-arginine is increased. Thereafter, NO can be combined with O_2_^−^ to form ONOO^−^ [22]. Increased NO levels generate H_2_O_2_ in mitochondria by cytochrome c oxidase inhibition [23]. In addition, nicotinamide adenine dinucleotide phosphate (NADPH) oxidase, cyclooxygenase, and electron transfer chain in mitochondria are sources for the increased intracellular O_2_^−^ [24–26].

For the protection of cellular homeostasis, there are several enzymes or other small molecules that act as oxidant scavengers and comprise the antioxidant defense system. The main antioxidant enzymes are (i) glutathione peroxidase (GPx) which catalyzes the conversion of H_2_O_2_ into water, (ii) superoxide dismutase (SOD) which converts O_2_^−^ to O_2_ or to the less reactive H_2_O_2_, and (iii) catalase (CAT) which also catalyzes the breakdown of H_2_O_2_. There are also other low molecular weight substances like ascorbic acid (AA), glutathione (GSH), and *α*-tocopherol. Among these, GSH is one of the most important redox buffers for the cells, since it can be found in all cell compartments. GSH/GSSG (glutathione disulfide, the oxidized form of GSH) is a good marker of oxidative stress. GSH can act as a cofactor for several enzymes, help in DNA repair, scavenge HO, H_2_O_2_, and lipid peroxides, and regenerate other antioxidants such as AA and tocopherols (Table 1).

Under physiological conditions, there is a balance between the formation of oxidant substances and their removal by antioxidant scavenging compounds [27]. Oxidative stress is the imbalance between antioxidant defense and generation of oxidants leading to enhanced oxidant concentration and constitutes a mechanism of injury for many disease processes [28]. The role of oxidative stress in the pathophysiology of several common conditions like diabetes mellitus, chronic heart failure, cancer, and degenerative, neurological, and autoimmune diseases is outside the scope of this review. Oxidative imbalance has been demonstrated in several sepsis studies. Takeda et al., in an early study, found an increased thiobarbituric acid reactive substance level in septic patients suggesting increased lipid peroxidation [29]. Decreased levels of antioxidants were also detected [30].

The clinical significance of oxidative stress in sepsis is demonstrated by several studies. Cowley et al. found that sepsis survivors had greater antioxidant potential than nonsurvivors and also that it was rapidly raised to normal or supranormal levels [31]. In two other prospective observational studies, total antioxidant capacity was correlated with Acute Physiology and Chronic Health Evaluation II (APACHE II) score [32] and the presence of a greater antioxidant deficiency correlated with mortality [33]. This deficiency was strongly indicated by two markers, GSH levels and CAT activity in erythrocytes, and persisted in time. Lower plasma vitamin C levels were detected in patients with multiorgan failure [34]. Harmful mechanisms of increased oxidants level in sepsis include modification of proteins, lipids, and nucleic acids contributing to cellular injury and endothelial dysfunction. In addition, the impairment of glycocalyx and the cellular junctions between endothelial cells lead to increased vascular permeability, a cornerstone of sepsis development [35].

## 3. NO and Cardiovascular Dysfunction

NO is produced from L-arginine by NOS [36], an enzyme with different isoenzymes (neuronal NOS or nNOS, inducible NOS or iNOS, endothelial NOS or eNOS, and mitochondrial NOS or mtNOS). iNOS produces NO in micromolar range as opposed to the other isoforms that produce NO in nanomolar range [37]. In sepsis, NO can be produced by several cells like activated macrophages, neutrophils, lymphocytes, and others [38–40]. Various molecules such as interferon *γ* (IFN*γ*), tumor necrosis factor a (TNFa), and interleukin 1*β* (IL-1*β*) involved in the septic inflammatory process are incriminated in the induction of NO production via iNOS hyperactivity. When the cell interacts with one of these molecules, IkB in the cytoplasm is degraded, NF-kB is permitted to move to the nucleus, and therefore expression of iNOS-associated genes is enhanced [41–43].

The effects of NO can be divided into effects on cardiac performance and effects on microcirculation. NO plays a pivotal role in vasodilation and vascular hyporeactivity to vasopressors. There are several studies that indicate this relationship. The injection of LPS in iNOS-deficient mice and the wild type as control provided the evidence that iNOS mediates impairment of vascular contraction [44]. Moreover, the inhibition of NO synthesis reversed shock in dogs induced by bacterial endotoxin [45] and also in septic rats by cecal ligation [46]. NO mediates negative inotropic effects to the cardiac function. LPS treatment of failing myocardium decreased maximum inotropic response to isoprenaline. The depression of cardiac contractility was attributed to enhanced iNOS activity and release [47]. In another study by the same investigators, the negative inotropic effect on human atrial and ventricular myocardium seemed to be mediated via generation of cyclic guanosine monophosphate (cGMP) [48]. On the other hand, other studies revealed that NO has no effect on the myocardium [49] or even that low concentrations of NO are preventive of cardiac performance. In a rodent heart model, coronary flow and ventricular function were reduced by LPS, effects that were partially prevented by supplementation of NO substrate, L-arginine. These improvements were partially blocked by the administration of selective iNOS inhibitors [50]. These data lead to the assumption that NO effects on cardiac performance are concentration dependent.

Other deleterious effects include protein nitrosylation and nitration, convertion of haemoglobin (Hb) to methaemoglobin (MetHb) which leads to red blood cell lysis and thus iron availability to the pathogens, and enhance the production of IL-6 and IL-8 and activation of NF-kB [26, 51] (Table 2).

## 4. Mitochondria and Apoptosis

**Figure 1:**
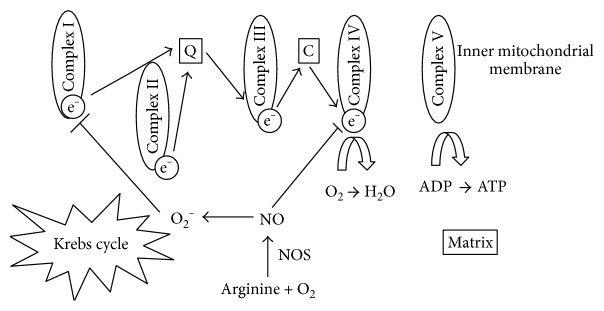
The mechanism of cytopathic hypoxia. The production of NO inhibits normal function of the respiratory complex IV interrupting the normal transport of electrons. O₂^−^ production is enhanced and reacts with NO inhibiting complex I normal function. NO, nitric oxide; NOS, nitric oxide synthase; ADP, adenosine diphosphate; ATP, adenosine triphosphate.

Mitochondria play a key role in redox dysregulation being at the same time sources and targets of oxidants. Oxidative phosphorylation takes place in the inner mitochondrial membrane where electron transport chain lies, consisting of five respiratory complexes (I–V). Electrons are transferred from one to another (I–IV) leading to adenosine triphosphate (ATP) generation in complex V (ATP synthase). Molecular oxygen is the final receptor of the electrons, and thus, an assessment of mitochondrial function can be performed through the measurement of oxygen consumption. The association between mitochondrial dysfunction and sepsis severity is addressed in several studies. In a fundamental one [52], skeletal muscle biopsies on 28 septic patients showed that nonsurvivors had lower ATP concentrations. Furthermore, vasopressor requirements were proportional to NO production as it was gauged by nitrite/nitrate concentrations and inversely correlated to complex I activity. Decreased ATP concentration and mitochondrial activity were also found in other human or animal studies [53, 54]. The pathogenesis of mitochondrial dysfunction is probably complex. NO seems to play a pivotal role by inhibiting the normal function of the respiratory complex IV. By binding to the specific complex, NO interrupts the normal transport of electrons and thus ATP production while at the same time the production of O_2_^−^ is enhanced. The generated O_2_^−^ reacts with NO leading to further mitochondrial dysfunction especially by complex I inhibition [55, 56] (Figure 1). The abovementioned mechanisms potentially explain the inability of the cells to utilize oxygen despite the adequate tissue oxygen tension. The term “cytopathic hypoxia” [57] refers to this phenomenon that eventually leads to multiorgan failure and worse outcomes. On the other hand, lower NO concentration seems to promote mitochondrial proliferation suggesting that NO effect on mitochondrial function may be concentration dependent [58].

**Figure 2:**
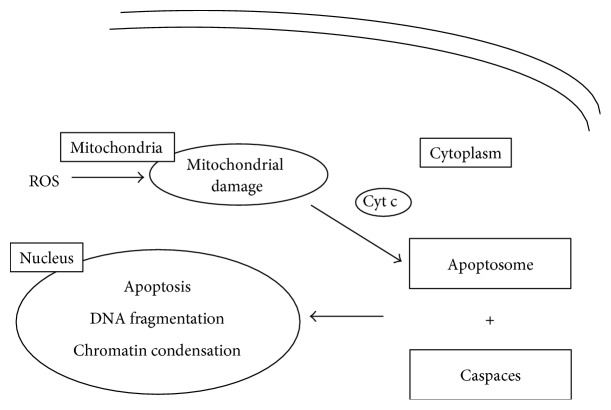
Mechanism of apoptosis. Mitochondrial damage by ROS releases cytochrome C, which contributes to the formation of apoptosome. The reaction of apoptosome with caspaces initiates cell apoptosis via DNA fragmentation and chromatin condensation. ROS, reactive oxygen species; Cyt C, cytochrome C; DNA, deoxyribonucleic acid.

Other potential mechanisms involve protein production and apoptosis. The decreased ATP synthase gene expression and subsequently impaired protein production were demonstrated by the administration of LPS in humans [59]. Apoptosis is the programmed cell death and is involved in sepsis pathogenesis. It can be triggered in a cell through either extrinsic or intrinsic stimuli. Mitochondria play a role in both pathways but especially in the intrinsic one. Mitochondrial damage by ROS can release cytochrome c, the mediator in electron flow between complexes III and IV, to cytosol. The next step is the formation of the “apoptosome” which reacts with caspases initiating the apoptotic pathway via deoxyribonucleic acid (DNA) fragmentation and chromatin condensation [60–63] (Figure 2).

## 5. Potential Therapies

The mainstay of sepsis management is source control, antibiotic administration, and haemodynamic support, but the relationship between antioxidant status and sepsis outcomes sets also the rationale for the use of antioxidant substances for the treatment of sepsis. Several molecules and different strategies were used in a plethora of studies in the past years with sometimes conflicting results.

### 5.1. Selenium

Selenium is essential for the synthesis of antioxidant enzymes, like GPx, and is involved in redox signaling and other immune responses [64]. The rationale for selenium supplementation derives from the correlation between low levels of selenium and disease severity and worse clinical outcomes in critically ill patients [65]. In a single-center clinical trial conducted on 54 septic patients, high-dose selenium administration did not result in reduction of 28-day mortality but increased the activity of GPx. No effect on the level of inflammatory cytokines was noted. However, selenium administration was associated with reduced incidence of ventilator-associated pneumonia (VAP) [66]. Moreover, in a recent multicenter randomized controlled trial (RCT), high-dose intravenous administration of sodium selenite was combined with procalcitonin-guided antimicrobial therapy in order to improve sepsis outcome. Both interventions failed to improve 28-day mortality [67]. In the most recent meta-analysis [68] after the review of 21 RCTs, the investigators concluded that parenteral supplementation of selenium in critically ill patients as a single agent or combined with other antioxidants had no effect on mortality, infections, length of stay, or ventilator days. The only significant effect was the reduction of infections in patients that were nonseptic at the initiation of therapy. In conclusion, even if there is a rationale for selenium administration, clinical trials failed to demonstrate benefits. Further research may reveal new insights in the role of selenium in sepsis pathophysiology.

### 5.2. Vitamin C

AA is the redox form of vitamin C and acts as a natural antioxidant. Plasma AA in patients with multiorgan failure was significantly lower [34], whereas low concentrations were inversely correlated with increased lipid peroxides [69] a marker of increased oxidative stress. Results from animal models demonstrated that AA ameliorates edema and hypotension and improves arteriolar responsiveness and capillary blood flow [70–73]. Experiments in healthy volunteers after induction of systemic inflammation by low doses of *E. coli* endotoxemia revealed that the hyporeactivity can be corrected by high doses of vitamin C, suggesting that oxidative stress may represent an important target for inflammation-induced impaired vascular function [74]. In a phase I safety trial of intravenous AA in patients with severe sepsis, infusion was safe and well tolerated [75]. In a retrospective analysis of the combination of hydrocortisone, vitamin C, and thiamine for the treatment of severe sepsis and septic shock, hospital mortality was 8.5% in the treatment group compared to 40.4% in the control group (*p* < 0.001). The propensity-adjusted odds of mortality in the patients treated with the vitamin C protocol was 0.13 (95% CI 0.04–0.48, *p* = 0.02). The sequential organ failure assessment score (SOFA score) decreased in all patients in the treatment group with none developing progressive organ failure. The duration of vasopressors was also smaller for the treatment group [76]. The very promising results of this study render the need for prospective randomized trials imperative for the determination of the role of vitamin C in sepsis treatment.

### 5.3. N-Acetylcysteine (NAC)

GSH is an important molecule recognized not only as an antioxidant but also as a mediator of immune and inflammatory pathways. GSH function is potentially enhanced by the administration of NAC, which has also itself an antioxidant and immunomodulatory activity [77–82]. Studies in humans demonstrated that the administration of NAC can significantly increase hepatosplanchnic blood flow attributed to the increase of cardiac index [83] and can augment neutrophil phagocytosis in patients diagnosed with sepsis, systemic inflammatory response syndrome (SIRS), or multiple trauma [84]. On the other hand, there are studies that demonstrate no influence on outcomes and the level of cytokines [85]. Sometimes, sepsis-induced organ failure was even aggravated [86]. The conflicting results may be due to a limited number of patients. Findings need to be confirmed in larger clinical trials.

### 5.4. Mitochondria-Targeted Antioxidants

Several strategies were used in order to reduce oxidative stress generated in mitochondria. The ability of lipophilic cations to accumulate in the mitochondria makes them good candidates for clinical studies. MitoQ (ubiquinone attached to a triphenylphosphonium cation) has been shown to protect mammalian cells from hydrogen peroxide-induced apoptosis [87, 88]. In another study, the effects of MitoQ were tested at first in vitro in an endothelial cell model of sepsis and afterwards in vivo in a rat model of sepsis. In vitro, MitoQ decreased oxidative stress and protected mitochondria from damage as indicated by a lower rate of ROS formation and by maintenance of the mitochondrial membrane potential. In vivo, MitoQ treatment resulted in lower levels of biochemical markers of acute liver and renal dysfunction [89]. The hypothesis that the administration of MitoQ would prevent endotoxin-induced reductions in cardiac mitochondrial and contractile function was tested in adult rodents. Endotoxin induced reductions in mitochondrial state 3 respiration rates, the respiratory control ratio, and ATP generation. These effects were ameliorated in the MitoQ-treated animals [90]. There are other substances conjucated to triphenylphosphonium cation as well, like vitamin E (MitoVitE), or ebselen, a selenium-containing compound with peroxidase activity (MitoPeroxidase) [91, 92]. Despite their promising properties, data on human studies are lacking.

Another option is the use of SOD mimetics. SOD mimetic M40401 improved vascular reactivity to vasopressors, reduced cytokine production, and improved mortality in a rat model of septic shock [93]. The ability of another SOD mimetic, the MnIIITE-2-PyP5+, to enter the mitochondria in vivo at levels sufficient to exert its antioxidant action was established by another study in rats [94]. These results encourage the development of SOD mimetics as therapeutic agents for sepsis.

TEMPOL was also used in animal studies with promising results [95, 96], but human studies are lacking. Antiapoptotic properties and ROS scavenging may explain its beneficial action.

### 5.5. NOS Inhibitors

The crucial role of NO in sepsis development and organ dysfunction led to the implementation of therapeutic strategies capable of reducing NO levels. NOS inhibition can be nonselective or selective for iNOS, which is predominantly synthesized during inflammation. In animal studies, nonselective NOS inhibition improved haemodynamics but increased mortality [97, 98]. The use of nonselective NOS inhibitors in patients with septic shock was terminated early because of increased mortality [99]. The inhibition of eNOS may explain the negative results of the study. The finding that the overexpression of eNOS is beneficial in septic animals [100, 101] led to the hypothesis that it is the excessive NO production by iNOS that is harmful and stimulated a research for selective iNOS inhibitors. Treatment with the selective iNOS inhibitor aminoguanidine inhibited the LPS-induced bacterial translocation by ameliorating intestinal hyperpermeability [102]. The rate of oxygen consumption was significantly restored in endotoxemic rats treated with aminoguanidine as compared with vehicle-treated endotoxemic rats [103]. Furthermore, in a porcine model of bacteremia where selective iNOS blockade was used, sepsis-induced plasma nitrate/nitrite concentrations were inhibited, hypotension was prevented without affecting cardiac output, and progressive deterioration in ileal mucosal microcirculation was blunted without mucosal acidosis [104]. An interesting alternative is ketanserin, a serotonin receptor antagonist. Several studies suggest that the administration of ketanserin is beneficial in septic animals. Mechanisms involved are the restoration of baroflex function [105] and the inhibition of iNOS expression via the MEK/ERK pathway [106]. The administration of ketanserin in septic patients resulted in improved microcirculatory perfusion assessed by direct visualization of the microcirculation with sidestream dark-field imaging [107]. The promising results deserve further evaluation in randomized trials.

### 5.6. Melatonin

Melatonin is the major hormone secreted by pineal gland predominantly at night. Melatonin has significant anti-inflammatory and antiapoptotic effects, but it can also act as an antioxidant scavenger for radical oxygen and nitrogen species [108, 109]. There are several animal studies depicting these beneficial antioxidant properties of melatonin in LPS or cecal ligation and puncture- (CLP-) induced septic shock [110–112]. Another important finding is the protection of mitochondrial dysfunction. Melatonin administration decreased mitochondrial NOS activity and inhibition of complexes I and IV in LPS-treated rats [113]. Furthermore, the results from another study suggest that melatonin can also prevent mitochondrial damage from the inducible isoform of mitochondrial NOS in septic mice [114]. Finally, it can restore mitochondrial production of ATP [115]. When healthy volunteers received melatonin before the administration of LPS, several markers of inflammation and oxidative stress were reduced [116]. In another study, melatonin treatment in septic newborns resulted in lower concentrations of lipid peroxidation products and other favorable outcomes [117]. In conclusion, melatonin has beneficial effects in sepsis that encourage the development of human studies since relevant data are lacking.

## 6. Conclusion

Oxidative stress mechanisms in sepsis are highly complicated. ROS and RNS play a pivotal role in sepsis evolution, but their specific role and importance remain obscure. Nevertheless, hyperpermeability, hypotension induced by reduced vascular tone, and mitochondrial impairment of respiration are key elements for multiorgan failure and thus mortality in septic patients. Several therapies were tested in clinical trials. Results are not sufficient for the implementation of these therapies in a clinical setting. An explanation may be that animal models do not completely resemble human sepsis. Further research is needed to answer questions about the underline mechanisms. Nevertheless, the increasing insight may alter our perception in sepsis development and management.

## Figures and Tables

**Table 1:** Summary of antioxidants and their effects.

Antioxidant	Mechanism of action
GPx	H_2_O_2_ to H_2_O
SOD	O_2_^−^ to O_2_
CAT	H_2_O_2_ to H_2_O and O_2_
GSH	Antioxidant scavenger, DNA repair, cofactor for enzymes
AA	Acts against oxidation of lipids, proteins, and DNA
*α*-Tocopherol	Scavenger for lipid peroxidation products

GPx: glutathione peroxidase; SOD: superoxide dismutase; CAT: catalase; GSH: glutathione; AA: ascorbic acid.

**Table 2:** Summary of NO effects in sepsis.

Positive effects of NO	
Preventive of cardiac contractility in low concentrations	
Mitochondrial proliferation	
Scavenger of oxygen free radicals	
Inhibition of oxygen free radical production	
Low pulmonary vascular tone	
Negative effects of NO	
Vasodilation/hyporeactivity to vasopressors	
Negative inotropic effect in high concentrations	
Inhibition of mitochondrial respiration	
Protein nitration/nitrosylation	
Methemoglobinemia	
Activation of NF-kB	

NO: nitric oxide; NF-kB: nuclear factor kB.
